# Applied Barcoding: The Practicalities of DNA Testing for Herbals

**DOI:** 10.3390/plants9091150

**Published:** 2020-09-04

**Authors:** Caroline Howard, Claire Lockie-Williams, Adrian Slater

**Affiliations:** 1 Biomolecular Technology Group, Leicester School of Allied Health Science, Faculty of Health and Life Sciences, De Montfort University, Leicester LE1 9BH, UK; 2 BP-NIBSC Herbal Laboratory, National Institute for Biological Standards and Controls, Potters Bar EN6 3QG, UK; Claire.Lockie-Williams@nibsc.org

**Keywords:** applied barcoding, *rbcL*, *matK*, *psbA-trnH*, nrITS, intron, *Hypericum*

## Abstract

DNA barcoding is a widely accepted technique for the identification of plant materials, and its application to the authentication of commercial medicinal plants has attracted significant attention. The incorporation of DNA-based technologies into the quality testing protocols of international pharmacopoeias represents a step-change in status, requiring the establishment of standardized, reliable and reproducible methods. The process by which this can be achieved for any herbal medicine is described, using *Hypericum perforatum* L. (St John’s Wort) and potential adulterant *Hypericum* species as a case study. A range of practical issues are considered including quality control of DNA sequences from public repositories and the construction of individual curated databases, choice of DNA barcode region(s) and the identification of informative polymorphic nucleotide sequences. A decision tree informs the structure of the manuscript and provides a template to guide the development of future DNA barcode tests for herbals.

## 1. Introduction

The use of DNA-based methods to identify herbal materials has been widely accepted as a complementary method to phytochemical and physical testing methods [1,2,3,4,5,6]. The ability of DNA-based methods to detect and identify contaminating materials has been its most beneficial feature, and it is this quality that sets these methods apart from others. The information provided by a well-performing, controlled and standardized genetic method enables producers, manufacturers and regulators to increase the quality of products, and prevent toxic materials from entering the supply chain [7].

The literature provides many examples of new methodologies, high sensitivity techniques and methods aimed at processed materials and degraded samples [5,8,9]. However, it is widely accepted that the most beneficial and appropriate application of DNA-based methods to the herbal industry will be upstream of production [7]. Ideally, these methods are applied to select seeds to be grown under conditions in line with cGACP guidelines. Although cultivated materials are preferred by the industry, a large proportion of medicinal plant material is still wild-harvested and this is arguably the most important area to which DNA barcoding methods should be applied [7]. Before any processing has occurred, DNA-based identification of the raw materials can provide a certainty of identity that is unparalleled, together with the detection of contaminating plant material.

As DNA barcoding projects have advanced, the steady accumulation of barcode sequences in public databases has proved to be a valuable tool for the design of DNA barcode authentication methods [10]. However, this wealth of information requires to be treated with caution to avoid poor quality and incorrectly labeled sequences. Furthermore, the abundance of sequences may create a level of “background noise” that can influence and misdirect the design of barcoding assays.

St John’s Wort (*Hypericum perforatum* L.) is one of the world’s leading herbal medicines in terms of global sales. This position in the marketplace has attracted the application of novel, DNA-based, identification techniques since 2004 [11], before the advent of plant DNA barcoding programs. Since then, the identification of medicinal plant material by DNA barcoding has been demonstrated for a significant number of species [1,3,4,5,6], including *H. perforatum* [12,13,14,15,16]. The DNA barcodes used for individual medicinal plants have generally been chosen on an ad hoc basis, depending on practical issues such as amplifiability and the need to discriminate between the target species and likely adulterants, rather than fundamental measurements of barcode effectiveness [17,18,19]. The reason for choosing one particular barcode region is often not made explicit in published work and may be the result of rather idiosyncratic decisions known only to the authors.

This manuscript provides a reasoned approach, informed by years of experience, toward applying DNA-based identification methods to a “target” commercial medicinal plant. A step-by-step process is described using St John’s Wort as a case study, providing a template that can be reproduced by researchers, companies and regulators looking toward the implementation of DNA-based methods for the first time.

## 2. Results

### 2.1. A Scheme for Applied Barcoding

**Figure 1:**
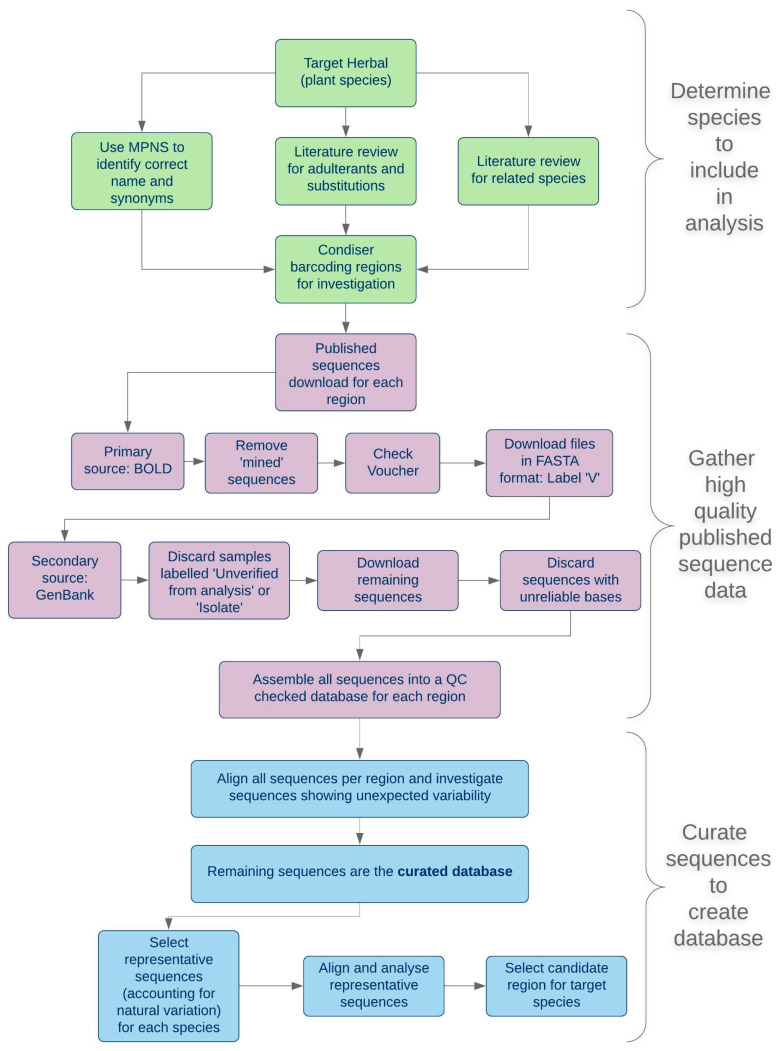
Flow diagram of DNA barcode region selection for discrimination of a commercial medicinal species from likely adulterant species. This shows a series of steps and decision points that can be used as a template to guide the choice of a suitable DNA barcode from published information through to laboratory analysis.

The choice of DNA barcode region and testing platform for a specified target herbal will depend upon practical considerations as well as more theoretical parameters of barcode efficiency. These include background information about the target species and known adulterants, the extent of DNA sequence data for different barcode regions and the availability of reference materials for test development. A flow diagram of a pragmatic approach to choosing a DNA barcode for herbal authentication is shown in Figure 1. Although an increasingly large number of DNA barcode sequences have been published, the quality and veracity of these are often questionable, and databases have numerous examples of sequences assigned to incorrect species [20,21]. For this reason, the decision tree in Figure 1 was developed to avoid some of the common pitfalls and provide a method to select high-quality sequences.

#### 2.1.1. Nomenclature, Taxonomy and Adulteration

The flow diagram proposes that three strands of background information about the medicinal plant of interest should be collated in parallel, prior to consideration of the DNA barcodes: current taxonomic information about the plant species and its relatives; confirmation of the correct botanical nomenclature and medicinal/common names, and investigation of adulteration issues and their underlying causes. Adulteration of herbals could arise from unintended contamination, misidentification during wild-harvesting, incorrect nomenclature, legitimate substitution or deliberate fraud.

There is considerable taxonomic information available about the genus *Hypericum* from both morphological studies captured in Robson’s monograph [22], and complementary chemical [23,24] and molecular studies based primarily on the ITS region [25,26,27]. This information immediately indicates a problem for DNA barcoding—this is a very large genus comprising some 490 species assigned to 36 Sections [22,28]. However, the main objectives of DNA barcoding a commercial medicinal plant are to successfully identify the target plant and to differentiate it from potential adulterants. The phylogenetic context of a plant provides valuable information about the relationships between target and potential adulterants, particularly the most closely related species. *H. perforatum* and its closest relatives are members of the Section Hypericum [29]. These “sister” species provide a “worst-case scenario” to test against, based on the presumption that being able to differentiate between the “target” and a “sister” species will be the most challenging objective. *H. maculatum* is the closest sister species to *H. perforatum*, grows in close proximity [30] and is a known adulterant [31], so the ability to discriminate between these two species can be regarded as a key requirement of the chosen barcode.

The nomenclature of *H. perforatum* and other members of the genus with medicinal properties can be confirmed at the Medicinal Plant Names Services (MPNS) [32]. This valuable resource lists botanical synonyms along with common and medicinal names. For example, *H. perforatum* L. is confirmed as the accepted name and attribution with the highest quality rating for confidence in the taxonomy, and three illegitimate scientific synonyms are noted. Issues around the nomenclature of *H. perforatum* subspecies are discussed in a separate MPNS publication [33]. There are 142 nonscientific names listed with the corresponding medicinal plant reference sources. Fifty medicinal reference sources citing *H. perforatum* are also listed. A number of other *Hypericum* species have medicinal properties and can also be found in this database (see Table 6).

There are several known adulterants of St John’s Wort herbal preparations within the genus *Hypericum* [31]. These range from close relatives which grow in similar habitats (e.g., *H. barbatum*, *H. hirsutum*, *H. maculatum*, *H. montanum* and *H. tetraptrum* in Europe [31]) to plants which grow widely in geographical areas where *H perforatum* is less common (e.g., *H. patulum* in India [34], *H. crux-andreae* in the New World [31] and possibly *H. undulatum* in China [35,36]).

The three strands of background information underpin the next stage, which is to consider possible DNA barcode regions. The initial literature review should include a search for prior recommendations of DNA barcodes for authentication of the target plant. There are relatively few published recommendations for DNA authentication of *Hypericum*, mainly focusing on the nuclear ribosomal internal transcribed spacer region (ITS) [11,12,13], but also *matK* [15].

#### 2.1.2. Selection of a Panel of Potential Adulterant *Hypericum* Species

The key function of a DNA barcode selected for authentication purposes is to discriminate between the correct “target” species and potential adulterant species. The barcode needs only to be unique for the target species. Provided all the nontarget barcodes differ from the target species, it is not essential for them also to differ from each other. Furthermore, the scope of relevant nontarget species can be restricted in order to dramatically simplify the problem. The underlying principle is that if the adulterants are known, it is unnecessary to be concerned with rare or geographically remote relatives of the target species [12] On this basis, a panel of 20 *Hypericum* species was selected comprising close relatives of *H. peforatum*, common commercial species and known adulterants (Table 6).

#### 2.1.3. Public Databases of DNA Barcode Sequences

The next stage is to search publicly available databases of existing DNA barcode sequences to create a collection for further study. The flowchart contrasts two major databases, describing the Barcode of Life Database (BOLD) [37] as the “primary source” and NCBI GenBank [10] as a secondary source, based on the reliability of source material identification and the accuracy of sequence data. The BOLD database is constructed specifically to hold complete records of DNA barcode projects. Full individual specimen records will include herbarium voucher details and photographs, collection details, DNA barcode sequence with supporting electropherogram traces for forward and reverse primers. However, BOLD also skims GenBank for barcode sequences, so it is important to differentiate full records from GenBank records. For example, there are 40 *rbc*L sequences for *H. perforatum* on BOLD but only 22 of these are full records with herbarium sheet photographs and sequence traces (Table 1).

One obvious point is that the more sequences are already publicly available (Table 1), the less de novo sequencing work is required in the laboratory. For example, despite the 40 *rbc*L sequences for *H. perforatum*, there is relatively poor coverage of the other panel species. In contrast, while there are also 40 ITS sequences available for *H. perforatum* between the two databases, there is also very good coverage of the other panel species. There are relatively few *psbA-trnH* sequences for most individual species, but the coverage of the panel species is nearly complete. On the other hand, *matK* is very poorly represented in either database. The prior selection of a panel of nontarget plants allows a manageable number of barcode sequences to be collected from the public databases, resulting in the creation of a small database of representative panel barcode sequences that can be analyzed and manually curated.

When these data are in place, a multiple alignment of each region will allow the level of intra- and interspecific variation to be determined. Having multiple accessions of each species at this stage may allow unreliable accessions to be detected and rejected, and consensus or representative sequences of each species to be selected to form the panel database. The objective at this stage is the identification of barcodes that show potential for defining a species-specific reference sequence against which to match test samples. Where there are gaps in the available published data, there will remain a question mark over the applicability of a particular barcode region—this is acceptable and can be addressed further on in the process. The practicalities of each region may be very different from one target species to another and should be confirmed experimentally.

#### 2.1.4. Reference Samples for Pilot Studies and Assay Standards

Having identified the panel species, a reference sample set must be established, and each sample verified as a true example of the species that it is intended to represent. Many laboratories will not have the resources or expertise to collect sufficient numbers of verified reference specimens, but may be able to find the relevant species represented in the DNA Bank schemes [38,39,40] operated by various botanical gardens, with DNA samples available from vouchered herbarium specimens. Once assembled, this reference sample set will provide a “proof of concept” study for candidate barcode regions.

Reference DNA samples of this type were obtained for most panel species (Table S1). The ease of amplification of each barcode region with standard primers differs between species, and this can only be measured experimentally. The amplification of each barcode region also serves to produce sequence data for any species within the reference sample set that was not available in public databases. These data can then be added to the original panel database and provide further validation of the selected barcode region.

#### 2.1.5. Test Samples for Barcode Assay Validation

A set of samples of *H. perforatum* and its closest sister species, *H. maculatum* were collected from different geographical locations around Lithuania (Table S2, Figure S2). These were used as a set of “unknown” samples to confirm the ability of each barcode to successfully distinguish *H. perforatum* samples from their closest relative. Once a set of test criteria were set for chosen barcode regions, these samples were used as “unknown” samples to determine whether the barcode test agreed with the botanical identification, and whether there was consistency between the barcodes.

### 2.2. The nrITS Barcode Region

#### 2.2.1. A Curated Database of ITS Sequences

The availability of published *Hypericum* ITS sequences from 2004 [11] was a major pragmatic reason for the authors choosing this barcode region for developing authentication tests from 2007 onwards. Since then, the number of *Hypericum* sequences available has grown considerably as the data from large-scale phylogenetic studies have been deposited in GenBank. One problem with this abundance of information is that the length and quality of accessions and the reliability of their original identification is inconsistent. As a result, a BLAST search of the database with a genuine *H. perforatum* ITS sequence will often include sequences from a number of different species in the top 50 hits, some having higher scores than genuine *H. perforatum* accessions. In order to filter this “background noise” effect, irrelevant and unreliable sequences were identified using the BLAST distance tree facility to identify and discard obvious outliers (typically singleton accessions with less than 95% identity to any other *H. perforatum* sequence) [20,21]. Tightly clustering groups of sequences unique to individual species (particularly vouchered specimens from a variety of sources) were collected and aligned. Where a small number of sequences showed near to 100% identity, a representative accession was chosen for the curated database. Species represented by larger numbers of accessions were typified by highly similar sequences with infrequent polymorphisms in individual accessions. In these cases, an artificial consensus sequence was constructed to represent the species. Where there were sufficient accessions to detect consistent patterns of variation within the species, consensus sequences representing each group of variants were designed, resulting in three *H. perforatum* ITS subtypes and two for *H. maculatum*. The ITS curated database is shown as a multiple alignment in Figure S1.

The alignment highlights several points of wider relevance. One is the clustering of regions of variation between species into “hotspots” within the ITS1 and ITS2 regions [13]. (The 5.8S coding sequence between the ITS1 and ITS2 regions is completely conserved.) These hotspots are ideal locations in which to detect “mini-barcodes” for targeting by PCR primers [12,13,14,41]. However, the main boundaries of variation at these hotspots can be seen to fall between Sections or larger clades. In consequence, there is very little variation between the members of the Section Hypericum, and most particularly between *H. maculatum* and *H. perforatum*. There are very few positions where both *H. maculatum* subtypes consistently differ from all three of the *H. perforatum* subtypes. Position 87 is unique to *H. perforatum*, position 467 is unique to *H. maculatum*, and 658/9 are not unique to either, but discriminate between the two species.

An advantage of starting with a large sequence dataset is that polymorphisms specific to one or other subtype of *H. perforatum* or *H. maculatum* can be detected. Limited sequence data might lead to the choice of these subtype polymorphisms as key determinants of a target or adulterant species, with subsequent false-positives or negatives. For example, a limited set of *H. perforatum* sequences that were all subtype I might indicate that the polymorphism at position 10 was a unique species marker of *H. perforatum*, but then give false negatives for any subtype II and III *H. perforatum* samples.

#### 2.2.2. ITS Barcoding of Reference Samples

The ITS region of all the reference DNA samples was amplified and sequenced successfully, apart from the. *H. delphicum* sample 13938. Alignment of the sequences with the *Hypericum* panel ITS database showed that each reference species ITS sequence had the closest match (at least 99% identity) to the corresponding panel sequence, apart from *H. perforatum* sample 13876 (due to a poor quality sequence) and *H. maculatum* sample 13896 (which was not a close match to the reference *H. maculatum* sequences and showed closer resemblance to members of the Section Ascyreia).

### 2.3. The ITS2 Barcode Region

#### 2.3.1. ITS2 Sequences

The ITS2 subregion of ITS has been championed as the ideal barcode for medicinal plants [42,43,44]. Separate ITS2 barcoding projects have been conducted, while ITS2 sequences can also be extracted from full ITS accessions. The ITS2 region seen in Figure S1 contains sufficient variation to differentiate the panel members from *H. perforatum* but it is inevitably more restricted than using the entire ITS region.

#### 2.3.2. Secondary Structure of ITS2 Sequences

**Figure 2:**
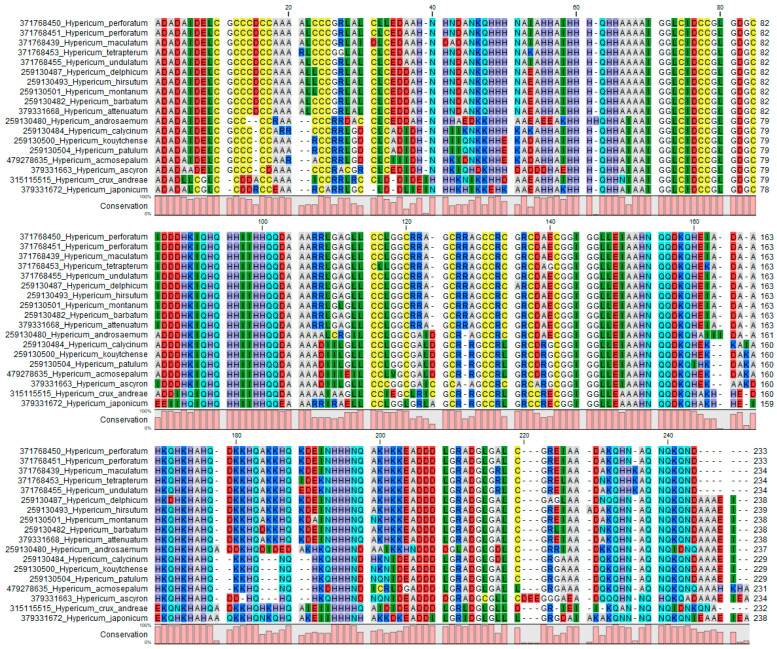
Alignment of panel species ITS2 sequences with RNA secondary structure represented by a single letter (pseudo amino acid) code [47].

One advantage of the ITS2 region is that it is possible to predict the secondary structure of the transcribed spacer RNA and use this information to support one-dimensional sequence alignments [45,46]. The ITS2 Ribosomal RNA database contains direct fold and homology modeled secondary structures for many animal and plant species, including 18 of the *Hypericum* panel. These models were aligned using a secondary structure as well as sequence information (Figure 2).

The alignment of sequence plus secondary structure ensures that the alignment is more robust and base-pairing information from the RNA structure provides additional points of difference between species. For example, *H. perforatum* differs from *H. maculatum* at positions 31, 41, 219 and 235 and 236 (Figure 2), whereas a simple sequence alignment shows only three SNPs (Figure S1)

It is possible to analyse complementary base pair changes (CBCs) between ITS2 sequences. These are polymorphic sites which are complemented by a second polymorphism to maintain base-pairing in stems of the RNA secondary structure. These have been hypothesized to follow species boundaries [48,49,50], but analysis of the number of CBCs between *H. perforatum* and the panel species indicates that they tend to map the clades defined by Meseguer et al. [26] (Table 2). Thus, there are no CBCs that distinguish any of the Section Hypericum or the related Sections in clade E. The species in clade D show one CBC, clade C has two CBCs and B has 4–5 CBCs. The one anomaly is that *H. ascyron* in the Section Roscyna in clade D has five CBCs. This matches the large number of differences seen in the primary sequence between this species and the rest of the panel, which cast doubt on the original botanical identification of these accessions.

### 2.4. The rbcLa Barcode Region

#### 2.4.1. A Curated Database of *rbcLa* Sequences

As shown in Table 6, there are a limited number of *rbc*L sequences in the BOLD database and not many more in GenBank, apart from *H. perforatum*, which is well represented. The alignment of these sequences shows there to be very little variation between species, particularly those within one Section or closely related sections. A limited haplotype map was constructed, indicating SNPs that were present at the same position in all the accessions from more than one species. Five positions were mapped (180, 300, 372, 379, 492) where the patterns of coinheritance effectively split the panel into two main haplotypes: 1 and 2 (Table 3).

There were sufficient *H. perforatum* accessions to detect two species-related SNPs. The T at position 66 was found in all of the *H. perforatum* accessions, while the T at 263 was found in a large majority. Neither SNP was found in any other accession, particularly the eight *H. maculatum* BOLD accessions. This specific haplotype pattern was designated type 1p. There appeared to be several other species-related SNPs in the *rbc*L barcode, but these are not directly relevant to *H. perforatum* authentication and would require more sequence data to ascertain their significance.

#### 2.4.2. Haplotype Mapping of Reference Samples

Using the haplotype map defined from the panel, it was possible to assign the reference samples to haplotypes 1 or 2 (Table 4). Several samples showed an intermediate haplotype not observed in any of the panel accessions, with the three 5′ SNPs matching haplotype 2 and the two 3′ SNPs matching haplotype 1. These were designated as type 1–2.

Only one of the *H. perforatum* reference standards perfectly matched the 1p haplotype (*H. perforatum* 13876). The other two *H. perforatum* samples (13921 and 13932) had a type 1–2 haplotype with neither *perforatum*-related SNP present. Other anomalies included the *H. maculatum* reference with a type 2 rather than type 1 haplotype and the *H. androsaemum* reference with a type 1–2 rather than type 2 haplotype.

### 2.5. The matK Barcode Region

As noted in Table 1, there are very few *matK* sequences deposited in either BOLD or GenBank. In this situation, it would be necessary to rely on the *matK* sequences obtained from reference samples in the laboratory. However, the *matK* barcode proved to be quite intractable with regard to amplification and sequencing. Several different published primer pairs were tested (see Materials and Methods) but none had good success rates and only four of the 12 DNA standards were able to be sequenced. There was better success with the test sample collection (50% success rate for sequences obtained) but without a range of panel species sequences for comparison, this was regarded as insufficient to proceed.

### 2.6. The psbA-trnH Barcode Region

#### 2.6.1. A Curated Database of *psbA-trnH* Sequences

**Figure 3:**
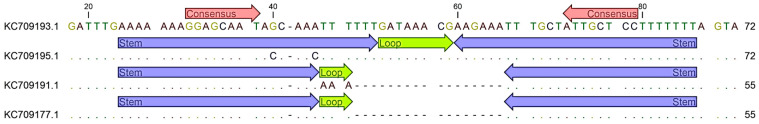
Alignment of selected *Hypericum* panel *psbA 3′* UTR sequences. The predicted stem-loop RNA structures and consensus sequences [52] are shown. KC709193, *H. olympicum*; KC709195, *H. hirsutum*; KC709191, *H. perforatum* C47; KC709193, *H. perforatum* C22.

There are only a few *Hypericum psbA-trnH* accessions in the BOLD database, but GenBank contains *psbA-trnH* sequences for a large number of *Hypericum* species from a large-scale taxonomic project [26]. Alignment of the panel sequences indicates some of the characteristic features of this region. The 3′ intergenic spacer region is characterized by A/T rich sequences with many homopolymeric stretches. Individual accessions of the same species showed random long insertions, making alignment difficult and raising doubts about the reliability of sequences from single accessions representing an entire species. There were, however, some characteristic features of *H. perforatum* that could be used to discriminate this species from all the others. Apart from three SNPs, there were two consistent insertions and a large deletion towards the 5′ end of the region. This deletion was found in all six of the complete *H. perforatum* accessions and in some but not all of the other Section Hypericum species. On closer inspection, this deletion was found to occur in the loop region of the characteristic stem-loop structure in the 3′ UTR of the *psbA* gene [52,53]. Figure 3 shows the sequence at the stem-loop structure in four of the panel sequences, two *H. perforatum* variants plus *H. olympicum* and *H. hirsutum*. The location of the consensus sequence described in [52] is shown along with the full stem and loop sequences relative to the deletion.

**Figure 4:**
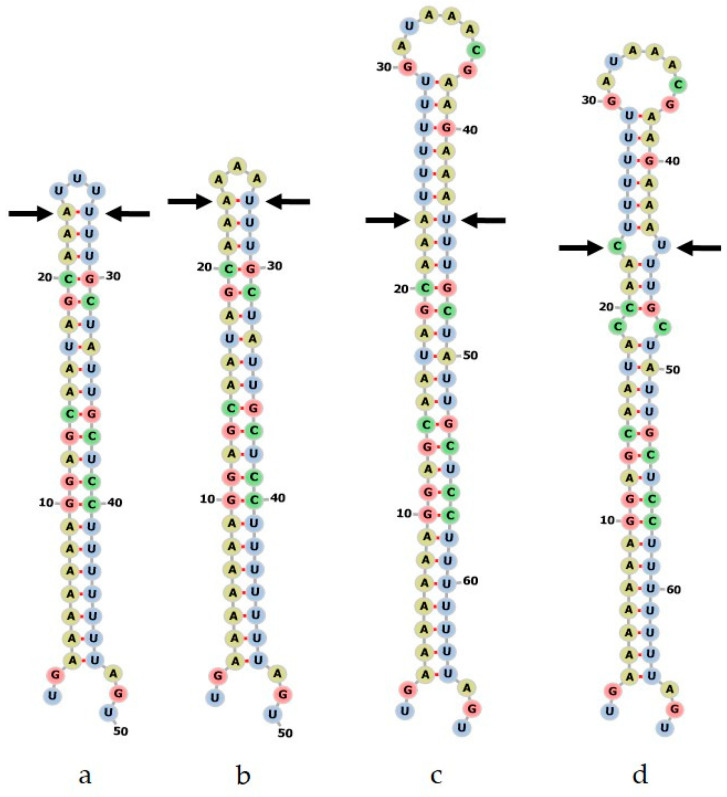
Predicted RNA secondary structures of *Hypericum psbA 3′* UTR sequences. a, *H. perforatum* C22; b, *H. perforatum* C47; c, *H. olympicum*; d, *H. hirsutum*. Arrows indicate the equivalent base pair in each structure.

The corresponding secondary structures are shown in Figure 4. The two *H. perforatum* variants can be seen to differ only in the three base loop sequence. The sequences lacking the deletion have an extended stem, with some variation in sequences creating interior loops.

#### 2.6.2. Reference and Test *psbA-trnH* Sequences

**Figure 5:**
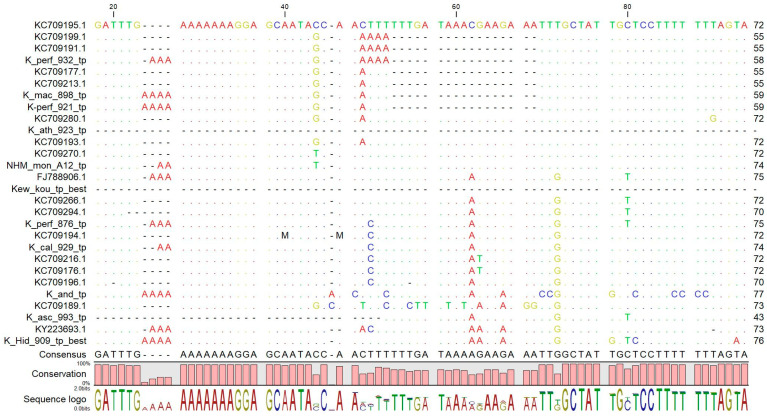
Alignment of database and reference sample psbA 3′ UTR RNA stem-loop region. Database sequences are labelled with GenBank accession numbers. Reference samples are labelled according to source (K = Kew; NHM = Natural History Museum), abbreviated species name (“perf” = *H. perforatum* etc.) and abbreviated voucher number as shown in Table S1.

The reference sample *psbA-trnH* sequences were compared with the database sequences (Figure 5). Most reference sequences matched the features observed in the corresponding panel sequence. Two *H. perforatum* reference samples (13921, 13932) had the characteristic truncated stem-loop (one with AAA in the loop, the other with UUU). In contrast, *H. perforatum* 13876 did not match this pattern and had an extended stem-loop structure. (Note—this is in contrast to the *rbc*L haplotypes, where 13876 had the expected haplotype 1p, but 13921 and 13932 were type 1–2).

### 2.7. Choosing the Optimal Barcode

Following the survey of suitable barcodes, it was clear that *matK* was not a suitable candidate barcode for two practical reasons: lack of database sequences and poor PCR amplification and sequence analysis. Of the remaining three, *rbc*L was shown to be a suitable discriminator at the genus and Section level, but the key challenge of differentiating between *H. perforatum* and *H. maculatum* was found to rest on just two SNPs. The *psbA-trnH* region showed a more suitable degree of variation between species, but evidence of frequent insertion/deletion events and homopolymer length heterogeneity within species would make the definition of a precise quality standard barcode difficult. The stem-loop region anchors a variable region between two conserved sequences, so could serve as a more useful indicator if this was shown to precisely map the species boundary.

**Figure 6:**
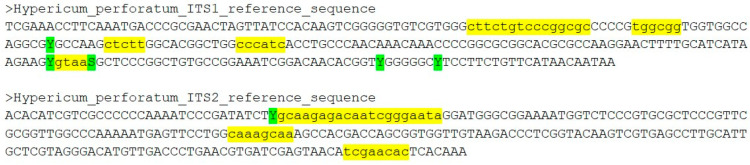
Proposed *H. perforatum* reference sequence from the ITS1 and ITS2 regions. Lower case bases (yellow highlight)—exact match required; Uppercase bases—exact match not required (95% identity required overall). Polymorphic positions in *H. perforatum* are shown with ambiguous single code letters (green highlight).

The ITS region starts with the advantage of a large database of sequences and sufficient variation to discriminate *H. perforatum* from all the other members of the panel. The ability to extract ITS2 sequences from the barcode and perform secondary structure analysis provides further confidence in the differentiation of *H. perforatum* and *H. maculatum*. A standard ITS reference barcode for *H. perforatum* for a pharmacopoeial monograph could comprise the two regions, one capturing the two main variable regions of ITS1, and the other covering ITS2 (Figure 6). The lowercase letters define exact bases that must be matched, while the uppercase bases show the remaining sequence [54,55]. There is an overall requirement for a 95% match between a test sequence and the reference, as well as an exact match with defined bases.

#### 2.7.1. ITS Barcoding of Test Samples

Based on this choice, a collection of *H. perforatum* and *H. maculatum* specimens from Lithuania were tested against the ITS reference barcode [56]. The ITS regions of the test sample collection were amplified and sequenced with a success rate of just over 70%. Eleven of the fourteen *H. perforatum* labeled samples matched the reference ITS barcode, while the remaining three did not meet the criteria and would be rejected in a quality control situation. None of the 12 samples labeled as *H. maculatum* matched the *H. perforatum* ITS reference barcode and showed closest similarity to *H. maculatum* ITS barcode sequences. (Table 5).

#### 2.7.2. Supporting Evidence from *rbc*L Haplotype and *psbA-trnH* Stem-Loop

The failure to amplify and sequence all of the test sample ITS barcodes was one reason to seek supporting evidence from the *rbc*L and *psbA-trnH* barcodes. These were amplified and characterized for their *rbc*L haplotype (see Table 3 and Table 4) and *psbA-trnH* stem-loop length (see Figure 3 and Figure 4). These characters are recorded in Table 5. Nearly all of the “perf” samples had the 1p *rbc*L haplotype, but the majority of “mac” samples also showed this haplotype. The apparent specificity of the 1p haplotype to *H. perforatum* was called into question by these results. Two samples showed an anomalous 1–2 *rbc*L haplotype (mac06 and perf02) despite having ITS sequences consistent with *H. maculatum* and *H. perforatum* respectively.

The psbA stem-loop structure showed a better correlation with the botanical identification and ITS sequences of samples. Of 22 nominal *H. perforatum* samples, the identity of 12 was supported by the ITS and *psbA* barcodes. Another five without ITS sequences were designated as likely *H. perforatum*, as was one of the *H. maculatum-*labeled samples. The identity of the remaining six remained undetermined, largely due to the failure of the ITS barcoding. The identity of 10 of the 16 *H. maculatum* samples was confirmed by ITS + *psbA*, with another five being consistent with *H. maculatum* based on *psbA* stem length.

## 3. Discussion

### 3.1. Applied Barcoding for Herbals

The flowchart/decision tree shown in Figure 1 distills many years of experience designing DNA barcode quality control tests for the herbals industry [7] and in developing reference barcodes for pharmacopoeial monographs [54,55]. The flow effectively starts with the question—what exactly is the “target”? Is it a single species or are several species legitimately traded under one common name? Is there confusion in the trade about the correct species name(s) and is the taxonomy clear? Is adulteration a problem, what are the causes and are the adulterant species known?

This background knowledge lays the foundation for the next stage, which is to identify a suitable “adulterant panel”. In this case study, a panel of 20 *Hypericum* species was chosen. This is larger than would be required for many herbals and reflects the large size of the *Hypericum* genus and the fact that several different adulterant *Hypericum* species are known or suspected [31]. Twenty *Hypericum* species in the panel proved to be manageable in terms of the manual collection of database sequences, sequencing of reference samples and interpretation of multiple sequence alignments. Careful consideration of the composition of the panel should ensure not only that all likely adulterant species can be differentiated from the target, but also that unknown adulterants will be highly unlikely to fortuitously match the *H. perforatum* reference barcode sequence.

For many herbals, there may just be one or a few problematic species to discriminate from the target plant. This obviously makes the design of the panel easier, though the process of choosing a suitable barcode may still depend upon the recognition of a small number of discriminatory polymorphisms if the adulterant is a close relative. There are situations where the known adulterant is not a close relative or may not even be congeneric [57]. If the aim is restricted to discriminating between a target and specific unrelated adulterants, finding a discriminating barcode should be straightforward, but it must be noted that the resulting reference barcode sequence will not necessarily be unique to the target species, nor indeed allow discrimination of any other adulterants.

### 3.2. Choosing a Barcode for Species Identification

The choice of a suitable authentication barcode will depend on several factors. At the DNA level, the key elements are the ability to discriminate between the target species and the adulterant panel, and the reliability of that discrimination. This discrimination will typically result from small regions of variation (mini- or microcodes [58,59,60]) that consistently map the species boundary between target and panel, rather than a calculation of overall genetic distances. Beyond the choice of the barcode sequence itself, the identification of variable minibarcode regions may also be an objective of the study in order to design authentication assays based on conventional, multiplex or qPCR primers [12,13,14]. For example, the qPCR primers designed to authenticate *H. perforatum* samples were designed using the same adulterant panel of *Hypericum* ITS sequences to target regions of essential bases (Figure 6) and tested against the same reference DNA collection [12]. The recent recommendations for validation of qualitative real-time PCR assays for diagnostic identification present a valuable opportunity to standardize such assays [61] but it should be recognized that such assays are a substitute for full-length DNA barcode identification, with the primers/probes acting as “minibarcode readers” [61]. The accuracy of such assays depends not only upon the discriminatory ability of the targeted minibarcode regions but also the specificity of design and performance of the primers/probes.

One factor that informs the choice of barcode is that the resultant identification assay should be fit for purpose and fulfill the needs of the industry and its regulators [7]. Chemical tests for herbal quality are typically based on simple HPTLC banding patterns rather than complex analytical profiles and our model for DNA testing has emulated this approach by picking out a small number of key informative SNPs. Indeed, the difference between a target and adulterant could be a single SNP if it is reliably known to be present in 100% of target samples and 0% of adulterant samples; this could still allow the design of a rapid high-resolution melt curve (HRM) authentication assay [62].

#### 3.2.1. Defining a Reference Barcode Sequence: Sensitivity and Specificity

As with any other diagnostic test, the reliability of a test based on matching a DNA barcode to a reference standard will depend upon the *specificity/sensitivity* of the reference barcode sequence and the *accuracy* and *precision* of the identification assay. The specificity of a reference standard barcode is measured by the proportion of false-positive identifications and is related to the number of characteristic features that discriminate between the target species and its adulterants. The sensitivity is a measure of the proportion of false negatives and reflects the extent to which intraspecific variation is captured within the reference sequence. The sensitivity and specificity of the reference sequence are therefore heavily dependent on the number and breadth of available target and adulterant sequences. The more barcode sequences collected from different laboratories and geographical locations, the more confidence can be placed in the barcode as a unique and consistent identifier of the target species. In this study, the ITS region had a starting advantage of having large numbers of *H. perforatum* sequences, a reasonable number from closest relative *H. maculatum* and a broad spread from other species (Table 1). This increased the confidence that the informative sites stipulated in the reference barcode were representative and characteristic of the species as a whole, and also allowed unreliable sequences to be identified and ignored. The flowchart emphasizes the value of checking the sources of database sequences, particularly those on GenBank [20,21], and where possible matching sequences against conspecific accessions.

The large number of *H. perforatum* ITS sequences also allowed consistent sites of intraspecific variation to be identified (Figure S1) [12]. Four subspecies of H. perforatum are recognized by Robson (*perforatum*, *chinense*, *veronense* and *songaricum*) [29] but are rarely recorded in the published literature or database accessions [33]. Molecular phylogenetic studies have characterized two distinct gene pools in European *H. perforatum* populations, though their relationship to the subspecies is not clear [63]. This highlights the need to collect as many sequences and reference samples as possible in order to ensure that any intraspecific variation that might legitimately be found in commercial trade is captured and incorporated into the reference barcode sequence using ambiguity codes (Figure 6). There is also a requirement to curate reference sequences, as sequence data steadily accumulate and new target plant populations reach the market. The possibility of the barcode becoming outdated is guarded against by regular iterations of the process described in Figure 1, and by an active stakeholder community using the reference barcode in routine testing protocols and reporting back anomalous results. This feedback system is already in place for other pharmacopoeial test methods.

#### 3.2.2. Matching a Reference Barcode Sequence: Accuracy and Precision

In a diagnostic test based on matching test sample barcode sequences to a reference sequence, the accuracy and precision of the process start with the “wet laboratory” procedures of DNA extraction, PCR amplification and DNA sequence analysis. A flow chart to ensure that good quality sequence data is obtained from test samples has been recommended, along with proposals for the optimal application of DNA testing in herbal drug supply chains as an upstream triage system to complement chemical testing [7]. The subsequent process of matching a test barcode sequence to a reference sequence determines the accuracy of the identification.

In the *Hypericum* examples described in this paper, the definition of reference barcodes and matching of samples has been largely conducted by manual inspection of pairwise or multiple alignments. In the case of *psbA-trnH*, the definition of a single characteristic feature (long/short 3′*psbA* RNA stem) and subsequent matching of test samples is straightforward. The *rbcL* 5-base core haplotypes with two possible *H. perforatum*-specific SNPs was also straightforward to recognize and read manually. The ITS barcode was more complex to analyse, both in terms of defining the reference sequence and then matching the test samples. Several polymorphic sites were recognized that discriminated between members of the Section Hypericum and other sections, but far fewer were found that differentiated *H. perforatum* from its closest relatives. There is only one SNP in ITS1 and three in ITS2 that consistently distinguish all of the three subtypes of *H. perforatum* from *H. maculatum*, compared to seven SNPs that characterize the three *H. perforatum* subtypes. This exemplifies the requirement for a diagnostic identification assay to specify essential discriminatory bases rather than a measure of overall genetic divergence.

This diagnostic approach to identification is analogous to a botanical key for identification of plants by matching distinguishing morphological features. A number of barcode identification algorithms based on diagnostic methods have been developed [64,65,66,67,68]. These recognize short sequence strings [68] (“diagnostic distinguishers” [67] or “distinguishing subsequences” [66]) in reference sequences which are then matched to query sequences. These are applicable to single barcode regions or entire genomes, and may be independent strings or located within their surrounding context [65]. Comparison of these diagnostic algorithms with pairwise distance [18,69], similarity, hierarchical clustering and phylogenetic tree-based methods indicates superior identification accuracy [64,65]. The reference sequence matching method described here is effectively a simplified diagnostic method in which the reference database is a single reference sequence and the diagnostic distinguishers are the prescribed essential bases. The requirement for overall similarity with the entire barcode sequence can then be viewed as contextualizing the diagnostic strings rather than using the similarity threshold to identify the species directly.

### 3.3. Recommended Barcodes for H. perforatum Authentication

The choice of ITS as the primary barcode was not dictated solely by the number of available sequences. The other barcodes showed suboptimal characteristics that have been noted for many other plant groups. The *rbc*L barcode had insufficient variation to discriminate between species or even Sections of the genus. The three haplotypes noted were based on five SNPs. In combination, these appeared to differentiate three clades containing species from related Sections, but the expectation that *H. perforatum* and *H. maculatum* samples would always show a type 1 haplotype was undermined by anomalous results obtained with three of the reference samples (Kew 13896, 13921 and 13932) and two of the test samples (mac06 and perf02). The two apparent *H. perforatum*-specific SNPs proved to be even more unreliable when tested against the Lithuanian collection, with the majority of *H. maculatum* samples showing the *H. perforatum* type 1p haplotype.

The *matK* region was ruled out for pragmatic reasons, as it proved difficult to amplify and sequence with several different published primer pairs. This may explain the paucity of published sequences which also weighed against the suitability of adopting this barcode for *H. perforatum* authentication. The third accepted plastid barcode, *psbA-trnH*, also showed traits that have been reported in other studies of this type. The barcode showed sufficient variation to distinguish between the panel species and *H. perforatum*, but even in this small sample set, there were several examples of random insertions and deletions within single accessions when compared to conspecific sequences. This behavior has been noted elsewhere [69,70,71] and a number of authors have focused on the specific features of the psbA 3′ UTR [52,53,71,72]. In this study, a shortening of the predicted RNA stem-loop structure in this region appeared to be characteristic of *H. perforatum* (though not unique to this species; Figure 3, Figure 4 and Figure 5). The obvious deletion in the *H. perforatum psbA* 3′UTR sequence could form the basis of a rapid authentication assay if shown to be reliable. Although anomalous results were found with the reference samples, this feature could discriminate between the “perf” and “mac” samples in the test collection with reasonable consistency (14/16 *H. maculatum* samples showed the long stem character, while 20/22 *H. perforatum* samples showed the short stem character). Interestingly, three of the “perf” samples that did not match the *H. perforatum* ITS reference (perf 03, 09 and 13) still showed the *psbA* short stem character.

### 3.4. Current Trends in Applied Barcoding

The disparities between the ITS, *psbA* and *rbc*L barcodes in certain specimens may reflect the complex apomictic and sexual reproduction systems in *H. perforatum* [63,73,74,75], its frequent hybridization with related species (*H. maculatum*, *H. undulatum* and *H. tetrapterum*) and variable ploidy [30,73,74,76,77,78]. These are all factors likely to contribute to lower levels of success in species discrimination in DNA barcoding studies [18] There is also the possibility of chloroplast capture leading to disparities between nuclear and plastid phylogenetic relationships [79,80,81,82,83]. One way to resolve this would be to sequence the entire plastid genomes of *H. perforatum* and related species with a view to discovering more effective genetic markers [84,85] or to use the entire plastid genome as a “super-barcode” [85,86].

Next-Generation Sequencing (NGS) technologies have been applied to herbal drug authentication using genome skimming or amplicon metabaroding approaches [1,8,87,88,89,90,91,92], including *H. perforatum* commercial products [16]. The quality of a herbal product is determined by the correct identity of its ingredients and its purity, measured by % contamination by a range of inorganic and biological contaminants. As described in this paper, DNA barcoding provides a direct method to confirm target species identity and. less frequently, to detect specific toxic adulterants [57,93,94,95,96]. A major advantage of metabarcoding assays is that they provide a “What’s In My Pot?” (WIMP) analysis [97] which determines both identity and purity. A key issue for the development of these assays is to meet the accuracy requirements of two quite discrete criteria: (i) the accuracy of the taxonomy prediction algorithms used in metabarcoding sequence analysis pipelines [98] and (ii) the quantitation of contamination by nontarget adulterants [6,99].

## 4. Materials and Methods

### 4.1. A panel of Potential Adulterant Hypericum Species

A panel of *Hypericum* species meeting some or all of the following criteria was selected:

1. Close relatives of *H. perforatum;*
2. Common in commercial trade as ornamental or medicinal plants;
3. Reported as adulterants of *H. perforatum*.

The selected panel comprized just twenty species (Table 6) and covers several close relatives from the Section Hypericum (*H. attenuatum*, *H. elegans*, *H. maculatum*, *H. tetrapterum*, *H. undulatum*), a number from the sections Ascyreia (*H. acmosepalum*, *H. calycinum*, *H. kouytchense*, *H. patulum*) and Adenosepalum (*H. athoum*, *H. delphicum*, *H. montanum*) and single representatives from the sections Androsaemum (*H. androsaemum*), Drosocarpium (*H. barbatum*), Myriandra (*H. crux-andreae*), Oligostema (*H. olympicum*), Roscyna (*H. ascyron*), Taeniocarpium (*H. hirsutum*) and Trignobrathys (*H. japonicum*) [12]. Table 6 indicates the criteria met for selection of each species as known or potential adulterants and/or close relatives.

### 4.2. Plant and DNA Materials

DNA reference samples were obtained from vouchered specimens available in The Royal Botanic Gardens, Kew, DNA Bank, https://www.kew.org/data/dnaBank/. A sample of H. montanum DNA was provided by Dr Mark Carine (The Natural History Museum, London). These are listed in Table S1. Dried leaf samples of *H. perforatum* and *H. maculatum* plants collected from the wild were used as the test collection (Table S2). Voucher specimens of each field accession are deposited in the Herbarium, the Institute of Botany/BILAS, Vilnius, Lithuania [56] and a map showing the location of their collection is shown as Figure S2.

### 4.3. DNA Extraction and Amplification of DNA Barcode Regions

DNA extractions were carried out using the Qiagen DNeasy Plant Mini Kit according to the manufacturer’s instructions, starting with 0.02 g dried leaf material.

PCR reactions consisted of Green GoTaq^®^ Flexi Buffer (Promega, Madison, WI, USA; 1×), MgCl2 (2.5 mM), GoTaq^®^ DNA Polymerase (Promega; 1.25 Units), relevant primers (0.1 μM each), dNTPs (0.1 μM each), and template DNA (0.7–1 μg) made up to a final volume 50 μL with nuclease-free water in 0.2 mL polypropylene tubes (Starlab, Milton Keynes, UK). The Applied Biosystems GeneAmp PCR System 9700 thermal cycler (Applied Biosystems, Foster City, CA, USA) was used with differing programs (Table 7).

Reactions without template DNA were utilized as controls. PCR products were run on 3% (*w/v*) agarose, 0.5 × TBE gels with 2 μL SYBRsafeTM (Invitrogen, Carlsbad, CA, USA) DNA stain at 90 V for ~30 min and analyzed in a BioRad Illuminator with ChemiDocXRS Camera and Quantity One software [100].

The PCR primers and conditions used to amplify each barcode region are shown in Table 7.

**Table 7:** Primers and programs used for amplification of barcode regions.

Region	Primer Sequence	Program
**ITS**	ITS1- TCCGTAGGTGAACCTGCGG ITS4-TCCTCCGCTTATTGATATGC [101]	7 min at 95 °C initial denaturation step, 30 cycles consisting of 1 min at 95 °C, 30 s at 60 °C and 1 min at 72 °C, final extension period of 7 min at 72 °C.
**trnH-psbA**	trnHf_05-CGCGCATGGTGGATTCACAATCC psbA3_f–GTTATGCATGAACGTAATGCTC [102]	5 min at 95 °C initial denaturation step, 35 cycles consisting of 1 min at 95 °C, 30 s at touchdown temperature and 1 min at 72 °C, final extension period of 7 min at 72 °C. Touchdown temperature began at 58 °C, reduced by 1 °C per cycle until 48 °C, then continued at 48 °C for the remainder of the program.
**rbcL**	rbcLa_f -ATGTCACCACAAACAGAAAC rbcLa_rev-GTAAAATCAAGTCCACCRCG [103]	5 min 95 °C initial denaturation step, 35 cycles consisting of 30 s at 95 °C, 20 s at 52 °C and 50 s at 72 °C, with a final extension period of 5 min at 72 °C.
**matK**	390F-CGATCTATTCATTCAATATTTC 1326R–TCTAGCACACGAAAGTCGAAGT 2.1-CCTATCCATCTGGAAATCTTAG 2.1a–ATCCATCTGGAAATCTTAGTTC X F- TAATTTACGATCAATTCATTC 5-GTTCTAGCACAAGAAAGTCG 3.2–CTTCCTCTGTAAAGAATTC 3F_KIM f-CGTACAGTACTTTTGTGTTTACGAG 1R_KIM r -ACCCAGTCCATCTGGAAATCTTGGTTC [104,105,106]	Initial “touch-up” program, 5 min 94 °C initial denaturation step, 5 cycles consisting of 30 s at 94 °C, 40 s at 44 °C and 40 s at 72 °C, followed by 30 cycles consisting of 30 s at 94 °C, 40 s at 46 °C and 40 s at 72 °C, with a final extension period of 3 min at 72 °C. The second amplification contained 2 μL of the initial PCR product diluted 1:200 as the DNA template. Second matK program: 5 min 94 °C initial denaturation step, 35 cycles consisting of 30 s at 95 °C, 20 s at 46 °C and 40 s at 72 °C, with a final extension period of 3 min at 72 °C.

### 4.4. DNA Sequence Analysis of Barcode Amplicons

Samples were either sequenced “in-house” or sent to an external sequence provider (Macrogen Europe B.V., Amsterdam, The Netherlands)

For in-house sequencing, preliminary PCR reactions were purified using QuickStep™ 2 PCR Purification Kit (EdgeBio, San Jose, CA, USA) and the DNA quantified using a Qubit^®^ Fluorometer and Quant-iT™ dsDNA BR Assay Kit (Invitrogen, Carlsbad, CA, USA).

Cycle sequencing reactions were conducted using the BigDye^®^ Terminator v3.1 Cycle Sequencing Kit (Applied Biosystems, Foster City, CA, USA). Reactions consisted of Ready Reaction Premix (2.5×; ABI), BigDye Sequencing Buffer (5×; ABI), sequencing primer (3.2 pM; VHBio, Gateshead UK or IDT, Leuven, Belgium) template PCR product (5–20 ng) and nuclease-free water. The sequencing program comprized; 1 min at 96 °C initial denaturation, 25 cycles consisting of 10 s at 96 °C, 5 s at 50 °C, 4 min at 60 °C. Extension products were purified using Performa^®^ DTR Gel Filtration Cartridges (EdgeBio, Maryland, USA), 10 μL Hi Di formamide was added and the sample thoroughly vortexed.

Products were analyzed on the ABI Prism™ 310 Genetic Analyzer (Applied Biosystems, Foster City, CA, USA), using a 47 cm capillary and Performance Optimised Polymer 6 (Applied Biosystems, Foster City, CA, USA). The run module used consisted of a 30 s injection at 2.0 kV, followed by electrophoresis running at 50 °C and 15 kV for 36 min. Sequence Analysis 5.2 (Applied Biosystems, Foster City, CA, USA) software was used to collect data, with Basecaller 310POP6, to create the output AB1 file.

### 4.5. Computer Analysis of Barcode Regions

Contig assembly of sequencing traces was performed using the CLC Main Workbench (Qiagen, Hilden, Germany). At least three reads in forward and reverse directions were assembled and conflicts resolved by manual inspection of traces.

Multiple alignment of sequences was also conducted on the CLC platform using the “Slow (accurate)” settings.

ITS2 secondary structure was analyzed using the tools available on the ITS2 database http://its2.bioapps.biozentrum.uni-wuerzburg.de/. The alignment of ITS2 sequences plus secondary structures was further analyzed and converted to single letter code using the 4Sale tools http://4sale.bioapps.biozentrum.uni-wuerzburg.de/.

The *psbA-trnH* secondary structure predictions were created with the RNAfold tools available at http://rna.tbi.univie.ac.at/cgi-bin/RNAWebSuite/RNAfold.cgi using the default minimum free energy (MEF) and partition function setting.

## Figures and Tables

**Table 1:** Availability of *Hypericum* panel barcode sequences in Barcode of Life Database (BOLD) and GenBank. Figures reported for BOLD represent the number of full accessions, with the total number, including those mined from GenBank, shown in parentheses. (Figures from March 2020).

	BOLD					GenBank				
Species	*rbc*L	matK	trnH-psbA	ITS	ITS2	*rbc*L	matK	trnH-psbA	ITS	ITS2
*H. acmosepalum*					1			1	5	
*H. androsaemum*	5 (6)	1			1	7	1	1	7	4
*H. ascyron*	1 (2)	1		1	2 (4)	5	3	1	16	3
*H. athoum*					1			1	4	
*H. attenuatum*					2				7	
*H. barbatum*					1				2	
*H. calycinum*	1	1			2	1	1	1	7	
*H. crux-andreae*						2		1	2	
*H. delphicum*					2				6	
*H. elegans*								1		
*H. hirsutum*	4 (5)		1	(1)	1 (3)	6		1	5	2
*H. japonicum*	2				3	9		5	10	7
*H. kouytchense*					2			1	4	
*H. maculatum*	7 (8)	(1)		1	1 (9)	11		3	14	3
*H. montanum*	2 (3)	1		1	2	3		1	6	1
*H. olympicum*					1			2	9	
*H. patulum*					2			1	8	2
*H. perforatum*	22 (40)	3 (6)	(3)	(1)	9 (27)	38	11	7	40	17
*H. tetrapterum*	2 (4)	1 (2)			4	3	2	2	5	2
*H. undulatum*	3 (4)	1			1	4	1	1	9	4

**Table 2:** Complementary base pair changes (CBC) in ITS2 RNA secondary structures, relative to the *H. perforatum* ITS2 secondary structure, calculated using the 4Sale program [48,51].

Species	Section	Robson Clade [24]	Meseguer Clade [26]	CBC/Perf
*H. delphicum*	Adenosepalum	27	E	0
*H. montanum*	Adenosepalum	27	E	0
*H. barbatum*	Drosocarpium	13	E	0
*H. attenuatum*	Hypericum	9	E	0
*H. maculatum*	Hypericum	9	E	0
*H. perforatum*	Hypericum	9	E	0
*H. tetrapterum*	Hypericum	9	E	0
*H. undulatum*	Hypericum	9	E	0
*H. hirsutum*	Taenocarpium	18	E	0
*H. olympicum*	Oligostema	14	E	0
*H. acmosepalum*	Ascyreia	3	D	1
*H. calycinum*	Ascyreia	3	D	1
*H. kouytchense*	Ascyreia	3	D	1
*H. patulum*	Ascyreia	3	D	1
*H. androsaemum*	Androsaemum	5	C	2
*H. crux-andreae*	Myriandra	20	B	4
*H. ascyron*	Roscyna	7	D	5
*H. japonicum*	Trignobrathys	30	B	5

**Table 3:** Haplotype map of the *rbc*L region, showing shared patterns of polymorphism between groups of species. Dots indicate compliance with the consensus sequences.

Species	Type	66	180	263	300	372	378	492
**Consensus**		**C**	**C**	**A**	**A**	**A**	**A**	**A**
*H. perforatum*	1p	T	T	T	G	C	.	.
*H. maculatum*	1	.	T	.	G	C	.	.
*H. tetrapterum*	1	.	T	.	G	C	.	.
*H. undulatum*	1	.	T	.	G	C	.	.
*H. montanum*	1	.	T	.	G	C	.	.
*H. hirsutum*	1	.	.	.	G	C	.	.
*H. androsaemum*	2	.	.	.	.	.	G	T
*H. ascyron*	2	.	.	.	.	.	G	T
*H. calycinum*	2	.	.	.	.	.	G	T
*H. crux-andreae*	2	.	.	.	.	.	G	T
*H. japonicum*	2	.	.	.	.	.	G	T

**Table 4:** Haplotype map of the *rbc*L region, assigning reference samples to haplotype. Type 1–2 represents an intermediate haplotype between types 1 and 2. Dots indicate identity with the consensus base at that position.

Reference	Type	66	180	263	300	372	378	492
**Consensus**		**C**	**C**	**A**	**A**	**A**	**A**	**A**
*H. perforatum* 13876	1p	T	T	T	G	C	.	.
*H. delphicum* 13938	1	.	T	.	G	C	.	.
*H. montanum* A12F	1	.	T	.	G	C	G	.
*H. kouytchense* 13866	1–2	.	.	.	.	.	.	.
*H. patulum* 13908	1–2	.	.	.	.	.	.	.
*H. perforatum* 13921	1–2	.	.	.	.	.	.	.
*H. perforatum* 13932	1–2	.	.	.	.	.	.	.
*H. androsaemum* 13854	1–2	.	.	.	.	.	.	.
*H. ascyron* 13993	2	.	.	.	.	.	G	T
*H. athoum* 13923	2	.	.	.	.	.	G	T
*H. calycinum* 13929	2	.	.	.	.	.	G	T
*H. maculatum* 13896	2	.	.	.	.	.	G	T

**Table 5:** Test plant samples of *H. perforatum* and *H. maculatum* [56]. The sample number indicates the preliminary botanical identification of the specimen. The primary identification is by matching the ITS sequence to the *H. perforatum* reference standard. Supporting evidence is provided by the *psbA-trnH* stem-loop (L = long; S = short; Sa = short with AAA loop) and *rbc*L haplotype (1(p) shows one *H. perforatum*-related SNP, 1p shows two SNPs). The final assignation (ID) is coded as M = *H. maculatum*; m = possible *H. maculatum*; P = *H. perforatum*; p = possible *H. perforatum*; U = undetermined.

Sample No.	DNA No.	ITS Match to *H. perforatum*	*trnH-psbA* Stem	*rbc*L Type	ID
mac 01	007	No	L	1	M
mac 02	011	No	L	1(p)	M
mac 03	012	No	L	1p	M
mac 04	018	No	L	1p	M
mac 05	030		L	1p	m
mac 06	045	No	L	1-2	m
mac 07	001		L	1	m
mac 08	031	No	L	1p	M
mac 09	027	No	L	1	M
mac 10	022		L	1(p)	m
mac 11	014		S	1p	p
mac 12	034	No	L	1p	M
mac 13	025	No	L	1p	M
mac 14	019	No	L	1	M
mac 15	036	No	s	1p	m
mac 16	023	No	L	1	M
perf 01	005		S	1p	p
perf 02	043	Yes	S	1-2	P
perf 03	017	No	Sa	1	U
perf 04	026	Yes	S	1p	P
perf 05	016		S	1p	p
perf 06	038		L	1p	U
perf 07	044	Yes	S	1p	P
perf 08	035	Yes	S	1	P
perf 09	029	No	S	1p	U
perf 10	015		S	1p	p
perf 11	032	Yes	S	1	P
perf 12	041		S	1p	p
perf 13	028	No	Sa	1p	U
perf 14	024		S	1p	p
perf 15	013		L	1p	U
perf 16	037	Yes	S	1p	P
perf 17	039	Yes	S	1p	P
perf 18	020	Yes	Sa	1p	P
perf 19	042	Yes	Sa	1p	P
perf 20	040	Yes	Sa	1p	P
perf 21	033	Yes	S	1p	P
perf 22	021		L	1(p)	U

**Table 6:** Hypericum species included in the restricted panel. The criteria for choosing these species are indicated in the columns: Rel, close relatives of *H. perforatum* in the Section Hypericum; MPNS, medicinal plants listed in the Medicinal Plants Names Service with at least one medicinal plant reference [32]; Cult, ornamental plants listed in the RHS horticultural database http://apps.rhs.org.uk/horticulturaldatabase/index.asp; Adult, plants identified as adulterants of commercial St John’s Wort herbal products [31].

Species	Attribution	Section	Rel	MPNS	Cult	Adult
*H. acmosepalum*	N.Robson	Ascyreia		-		Y
*H. androsaemum*	L.	Androsaemum		Y	Y	Y
*H. ascyron*	L.	Roscyna		Y		Y
*H. athoum*	Boiss. & Orph	Adenosepalum		-	Y	
*H. attenuatum*	Fisch. ex Choisy	Hypericum	Y	-		
*H. barbatum*	Jacq.	Drosocarpium		-		Y
*H. calycinum*	L.	Ascyreia		Y	Y	
*H. crux-andreae*	(L.) Crantz	Myriandra		Y		Y
*H. delphicum*	Boiss. & Heldr.	Adenosepalum		-		
*H. elegans*	Stephan ex Willd.	Hypericum	Y	Y		
*H. hirsutum*	L.	Taeniocarpium		-		Y
*H. japonicum*	Thunb.	Trigynobrathys		Y	Y	
*H. kouytchense*	H.Lev	Ascyreia		-	Y	
*H. maculatum*	Crantz	Hypericum	Y	Y		Y
*H. montanum*	L.	Adenosepalum				Y
*H. olympicum*	L.	Oligostema		-	Y	
*H. patulum*	Thunb.	Ascyreia		Y	Y	Y
*H. perforatum*	L.	Hypericum		Y	Y	
*H. tetrapterum*	Fr.	Hypericum	Y	Y	Y	Y
*H. undulatum*	Schousb. ex Willd.	Hypericum	Y			Y

## Supplementary Materials

The following are available online at https://www.mdpi.com/2223-7747/9/9/1150/s1, Figure S1: Multiple alignment of the ITS barcode region of *Hypericum* panel species. Table S1: List of reference DNA samples from DNA banks. Table S2: Collection of *H. perforatum* and *H. maculatum* plant leaf samples from Lithuania. Figure S1. Alignment of the adulteration panel of *Hypericum* ITS sequences.
