# Supplementary material for: Applied Barcoding: The Practicalities of DNA Testing for Herbals
Source: Plants (Basel). 2020 Sep 4;9(9):1150. doi: 10.3390/plants9091150 (PMC7570336; doi:10.3390/plants9091150)
Supplement: Supplementary file 1 [file plants-09-01150-s001.pdf]

Supplementary Information

Table S1. Reference samples from DNA Banks. **Kew**, Royal Botanic Gardens, Kew; **NHM**, Natural History Museum, London.

Source	Voucher Number	Species
**Kew**	13854	*H. androsaemum*
**Kew**	13993	*H. ascyron*
**Kew**	13923	*H. athoum*
**Kew**	13929	*H. calycinum*
**Kew**	13938	*H. delphicum*
**Kew**	13866	*H. kouytchense*
**Kew**	13896	*H. maculatum*
**NHM**	A12	*H. montanum*
**Kew**	13908	*H. patulum*
**Kew**	13876	*H. perforatum*
**Kew**	13921	*H. perforatum*
**Kew**	13932	*H. perforatum*

Table S2. Samples of *H. perforatum* and *H. maculatum* collected from Lithuania. The botanical identification is shown along with the wild collection location. Sample # refers to the dried leaf sample number assigned by the donors, DNA # is the randomized number assigned to DNA extracts by the authors.

Sample #	DNA #	Species	Habitat
**mac 01**	007	*H. maculatum*	Vilnius, Botanical Garden
**mac 02**	011	*H. maculatum*	Vilnius, Botanical Garden
**mac 03**	012	*H. maculatum*	Vilnius, Botanical Garden
**mac 04**	018	*H. maculatum*	Vilnius, Botanical Garden
**mac 05**	030	*H. maculatum*	Vilnius, Botanical Garden
**mac 06**	045	*H. maculatum*	Sirvintos, Ciobiskis
**mac 07**	001	*H. maculatum*	Raseiniai, Lyduvenai
**mac 08**	031	*H. maculatum*	Raseiniai, Lyduvenai
**mac 09**	027	*H. maculatum*	Raseiniai, Dubysa
**mac 10**	022	*H. maculatum*	Anyksciai, Svedasai
**mac 11**	014	*H. maculatum*	Kretinga, Kretinga
**mac 12**	034	*H. maculatum*	Kaunas, Girionys
**mac 13**	025	*H. maculatum*	Raseiniai, Katauskiai
**mac 14**	019	*H. maculatum*	Raseiniai, Gruzdiske

**mac 15**	036	*H. maculatum*	Silale, Pajuris
**mac 16**	023	*H. maculatum*	Raseminiai, Girkalnis
**perf 01**	005	*H. perforatum*	Kalvarija, Jungenai
**perf 02**	043	*H. perforatum*	Vilkaviskis, Naudsiai
**perf 03**	017	*H. perforatum*	Skudas, Mosedis
**perf 04**	026	*H. perforatum*	Anyksciai, Svedasai
**perf 05**	016	*H. perforatum*	Silute, Silute
**perf 06**	038	*H. perforatum*	Kaunas, Girionys
**perf 07**	044	*H. perforatum*	Svencioniai, Januliskis
**perf 08**	035	*H. perforatum*	Trakai, Rykantai
**perf 09**	029	*H. perforatum*	Palanga, Sventoji
**perf 10**	015	*H. perforatum*	Klaipeda, Karkle
**perf 11**	032	*H. perforatum*	Kelme, Tytuvenai
**perf 12**	041	*H. perforatum*	Svencioniai, Labanoras
**perf 13**	028	*H. perforatum*	Jurbarkas, Jurbarkas
**perf 14**	024	*H. perforatum*	Panevezys, Panevezys
**perf 15**	013	*H. perforatum*	Kedainiai, Kampai
**perf 16**	037	*H. perforatum*	Raseiniai, Ariogala
**perf 17**	039	*H. perforatum*	Jurbarkas, Skirsnemune
**perf 18**	020	*H. perforatum*	Neringa, Juodkrante
**perf 19**	042	*H. perforatum*	Raseiniai, Raseiniai
**perf 20**	040	*H. perforatum*	Prienai, Zarstai
**perf 21**	033	*H. perforatum*	Panevezys, Ramygala
**perf 22**	021	*H. perforatum*	Druskininkai, Latezeris

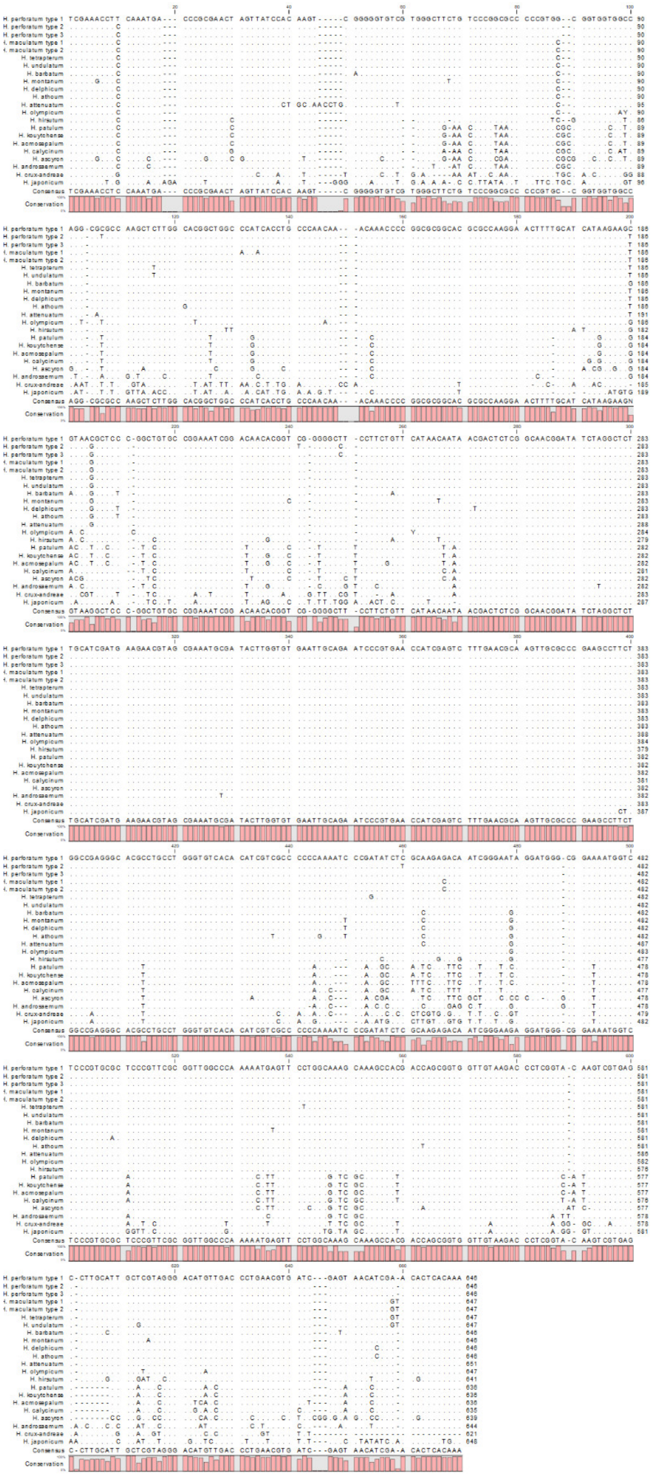

Figure S1. Multiple sequence alignment of the ITS region of the adulterant panel of *Hypericum* species. Three variants of *H. perforatum* and two of *H. maculatum* are shown aligned with ~~a~~ representative sequences of the other panel members.

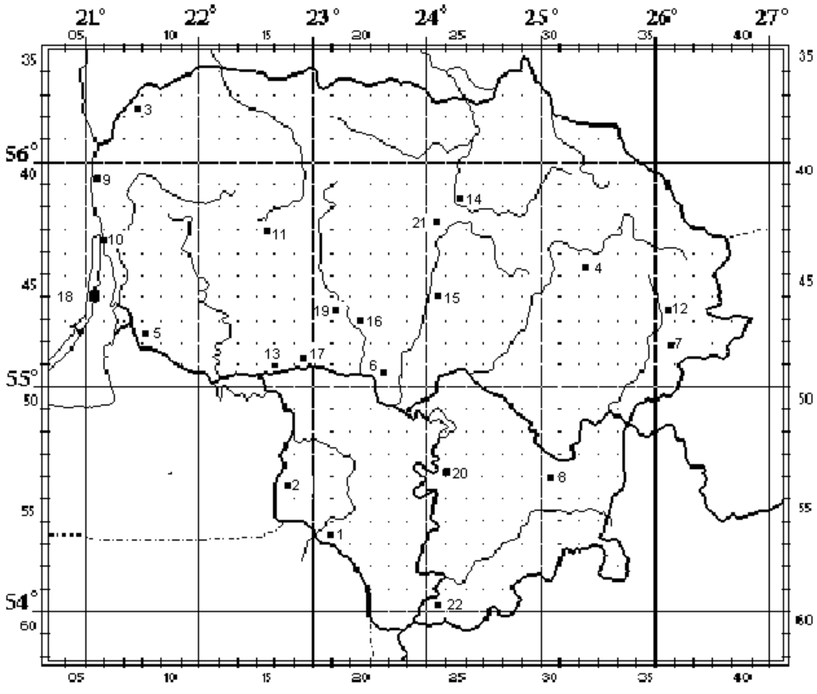

Figure S2a. Habitats of samples of *H. perforatum* from Lithuania. Numbers relate to samples in Table S2.

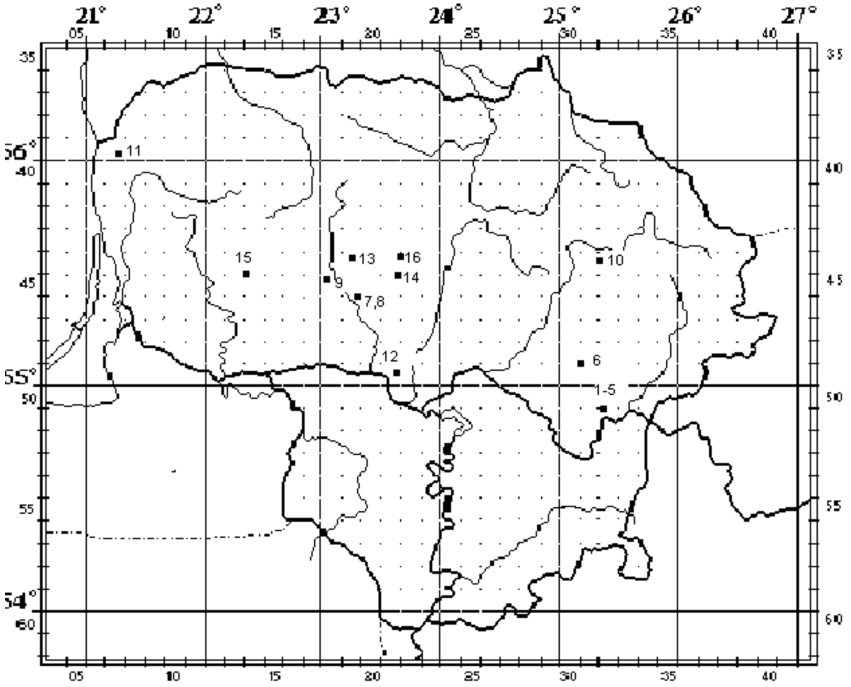

Figure S2b. Habitats of samples of *H. maculatum* from Lithuania. Numbers relate to samples in Table S2.
